# Long-Term Monitoring of Soil Microbiological Activities in Two Forest Sites in South Tyrol in the Italian Alps

**DOI:** 10.1264/jsme2.ME14050

**Published:** 2014-07-10

**Authors:** Rosa Margesin, Stefano Minerbi, Franz Schinner

**Affiliations:** 1 Institute of Microbiology, University of Innsbruck, Technikerstrasse 25, A-6020 Innsbruck, Austria; 2 Division Forestry, Autonomous Province of Bozen/Bolzano, Brennerstrasse 6, I-39100 Bozen/Bolzano, Italy

**Keywords:** forest soils, subalpine, submontane, soil microbiology, enzymes

## Abstract

We monitored microbiological properties in two forest sites over a period of 17 years (1993–2010) within the International Cooperative Programme on Integrated Monitoring of Air Pollution Effects on Ecosystems (ICP IM). The two study sites were located in South Tyrol in the Italian Alps at altitudes of 1,737 m a.s.l. (subalpine site IT01) and 570 m a.s.l. (submontane site IT02). Soil samples were collected in the late spring and autumn of 1993, 2000, and 2010, and were characterized by measuring respiration, key enzyme activities involved in the C, N, P, and S cycles and litter degradation, and the abundance of viable bacterial and fungal populations. Over the study period, an increase in mean annual air temperatures at both sites (+0.6°C and +0.8°C at IT01 and IT02, respectively) was calculated from trendlines. Significantly lower mean annual air temperatures, higher temperature fluctuations, and higher annual precipitation rates were observed at site IT01 than at site IT02. Subalpine site IT01 was characterized by significantly lower microbial activity (respiration, enzymes) and abundance than those at submontane site IT02. The year of sampling had a significant effect on all the parameters investigated, except for nitrification. Fungal abundance decreased consistently over the study period, while no consistent trend was noted among the other parameters investigated. Season only affected a few of the measured microbiological parameters: respiration and bacterial numbers were significantly higher in the spring than in the autumn, while the opposite was noted for xylanase and phosphatase activities. Soil fungi contributed essentially to xylanase and protease activities, while soil bacteria were mainly involved in degradation processes that required the activity of sulfatase.

Climate change is increasing surface temperatures worldwide. The European Alps already experienced an increase in the annual minimum temperature of approximately 2°C during the 20^th^ century (5). Mountain regions are especially vulnerable to climate change (12). In Alpine environments, climate change is expected to cause an upward migration of vegetation zones. This may alter the composition of vegetation as well as the quantity and quality of plant litter, which in turn affects the composition and functioning of microbial communities (14). Climate has one of the greatest effects on soil microbial activities (56).

Forests are expected to face significant pressures in the future from climate change. Data from long-term monitoring programs can be used to answer questions on the impacts of climate change and air pollution on forest ecosystems as well as the feedback of forests to the climate (9). Such a long-term monitoring program has been undertaken by the multi-disciplinary International Cooperative Programme on Integrated Monitoring of Air Pollution Effects on Ecosystems (ICP IM), which is part of the Effects Monitoring Strategy under the UN ECE (United Nations Economic Commission for Europe) Convention on Long-Range Transboundary Air Pollution (LRTAP) and became a permanent monitoring program from the beginning of 1993 (26). In this framework, integrated monitoring is carried out at 57 sites, mostly in Europe, in 23 countries.

ICP IM includes the monitoring of bioindicators that describe the state of the ecosystem (1). Since microbiological processes are central to forest growth, their monitoring constitutes an important tool for the characterization of forest soils (48). The ICP IM subprogram “Microbial Decomposition” is focused on monitoring microbiological parameters, with special consideration of soil respiration, microbial biomass, soil enzymatic activity, and N turnover dynamics (22, 53).

Soil biology is a significant component of soil quality, and microorganisms play vital roles in soil fertility and primary production through the decomposition of organic matter and nutrient cycling (2). Microbiological activity determines the net-mineralization of nutrients in an ecosystem; thus, any disturbance in ordinary activity will result in changes in both decomposition and nutrient uptake. Since the microbiological composition of soil is extremely difficult to monitor directly, indications and indirect biochemical measurements have to be applied (40). Soil biological properties, including soil respiration, soil biomass, microbial numbers, and enzyme activities, have been used successfully as biological indicators to evaluate soil metabolic activity and the effects of changing environmental conditions on soil ecosystems (17, 34, 38, 43, 45, 52). Soil (basal) respiration is considered to represent overall microbial activity that reflects the mineralization of organic matter in soil and is the most commonly used biological variable in soil studies, including forest ecosystems (53, 54). Soil enzyme activities are the direct expression of the soil community to metabolic requirements and available nutrients; the capacity of soil microorganisms to maintain soil enzyme functional diversity could ultimately be more important to the stability of an ecosystem than taxonomic diversity (10).

This study was performed within the ICP IM subprogram “Microbial Decomposition” and presented the microbiological characterization of two permanent monitoring sites (IT01 Ritten, IT02 Montiggl) located in South Tyrol in the Italian Alps that were established in 1992 as B class sites as classified by the ICP IM. The objective of this study was to monitor soil microbiological properties in the late spring and autumn of 1993, 2000, and 2010. Soil samples were characterized with regard to respiration, the key enzyme activities involved in the C, N, P, and S cycles, and litter degradation. Culture-dependent methods were used to obtain information on the abundance of bacterial and fungal populations. We evaluated the effects of the sampling year, season, and altitude (by comparing submontane and subalpine sites) on soil microbial activity at these sites. So far, the impact of global warming on microbial communities and activities in forest soils has been evaluated in short-term (2–3 years) soil warming experiments; however, no monitoring studies have been conducted for a period comparable to our 17-year study.

## Materials and Methods

### Description of the study sites

The two investigated sampling sites IT01 (Ritten) and IT02 (Montiggl) are two long-term monitoring sites that were installed in the Italian Alps in 1992 within the framework of ICP IM. These sites represent two widely distributed and forestally significant forest types in South Tyrol; their characteristics are shown in Table 1.

The subalpine site IT01 is located 7 km north of Bozen/Bolzano below the Rittner Horn at an altitude of 1,737 m above sea level (a.s.l.). The pedogenetic substratum consists of a mixed morainic and detritus cover of porphyritic pebbles and stones on quattziferous porphyry of the Permian period (Paleozoic). The soil was classified (FAO) as haplic podsol. The site consists of coniferous forests close to the timber line and is dominated by *Picea abies*, *Pinus cembra*, and *Larix decidua*. The climate is subalpine-continental with a mean annual temperature of 4°C and mean annual rainfall of 1,000 mm. Before confining the study site, high stress factors arose from grazing and touring (7).

The submontane site IT02 is located 8 km south of Bozen/Bolzano above the Small Lake Montiggl at an altitude of 570 m a.s.l. The pedogenetic substratum consists of colluvial material on quartziferous porphyry, which often comes to the surface in outcrops. The soil was classified (FAO) as dystric cambisol. The site consists of deciduous forests and is dominated by *Quercus pubenscens*, *Q. robur*, *Fraxinus ornus*, and *Ostrya carpinifolia*. The climate is mild continental with submediterranean influences, with a mean annual temperature of 11°C and mean annual rainfall of 900 mm. Before confining the study site, high stress factors resulted from grazing, touring, clear-cutting, and reforestation with allochthonous species (*Picea abies*) (7).

### Climatic conditions

Air temperature at the two sites was constantly monitored according to WMO standards at open field stations using the sensor t026 TTEPRH (Siap+Micros, Italy). Bulk precipitation was monitored by open field pluviometers using the sensor t027 TP500 (Siap+Micros, Italy).

### Soil sampling and sample preparation

In each site, 10 sampling spots that were distributed uniformly over the site were chosen in 1992 when the sites were established. Soil samples (*ca.* 5 kg) were collected from each of these sampling spots from the A_h_ horizon (top 10 cm). Sampling occurred in 1993, 2000, and 2010. To determine the effects of season, soil samples were collected in each sampling year from each site both in the late spring (May) and autumn (October). Immediately after sampling, soil samples were transported in cooled boxes to the laboratory, sieved (<2 mm), and immediately analyzed or stored at 2°C. Each soil sample was analyzed with three replicates.

### Physical and chemical soil properties

Soil samples were analyzed after sampling in the spring and autumn for dry mass (24 h at 105°C), maximum water-holding capacity, and soil organic matter (SOM; loss on ignition after 3 h at 430°C) as described (45, 46).

### Soil microbial activities

Soil respiration (basal respiration) was determined according to the ICP IM Manual (20). Soil samples were pre-incubated for 12 d at 50% of the maximum water-holding capacity to stabilize basal respiration. Soil samples were then incubated in air-tight 1-L vessels that contained NaOH solution to absorb the CO_2_ produced during incubation. After 18 h of incubation at a temperature of 22°C, BaCl_2_ was added and excess NaOH was titrated with HCl to the endpoint of phenolphthalein. Substrate-induced respiration (SIR) was determined using the same method in soil samples that were amended with glucose and incubated at 22°C in bottles containing NaOH.

Soil dehydrogenase activity was examined using trinitrophenyltetrazolium chloride (TTC, 1.5%, w/v) as a substrate and 0.1 M Tris-HCl, pH 7.8, as a buffer. After 16 h of incubation at 25°C, the produced triphenyl formazan (TPF) was extracted and measured spectrophotometrically (51, modified according to reference no. 45).

Soil xylanase activity was assessed using xylane (1.2%, w/v) as a substrate and 2 M sodium acetate-acetic acid buffer, pH 5.5. After 24 h of incubation at 50°C, the reduced sugars released during the incubation period were determined colorimetrically (45).

Protease activity was determined using casein (2%, w/v) as a substrate and 0.05 M Tris-HCl, pH 8.1, as buffer. After 2 h of incubation at 50°C, the amino acids released during incubation were extracted and quantified colorimetrically (27, 45).

Phosphomonoesterase activity (acidic phosphatase) was measured as described in the ICP IM Manual (20) by using 0.115 M *p*-nitrophenyl phosphate as a substrate and 0.5 M sodium acetate-acetic acid buffer, pH 5.0. After 1 h of incubation at 37°C, the *p*-nitrophenol released during the incubation was extracted and colorimetrically determined.

Arylsulfatase activity was determined using 0.02 M *p*-nitrophenyl sulfate as a substrate and 0.5 M sodium acetate-acetic acid buffer, pH 5.8. After 1 h of incubation at 37°C, the *p*-nitrophenol released during the incubation was extracted and colorimetrically determined (50, modified according to reference no. 45).

Nitrification was determined after the incubation of soil samples amended with ammonium chloride solution for 2 weeks (samples from site IT02) or 3 weeks (samples from site IT01 were incubated for longer due to a higher SOM content) at 25°C and the released amounts of ammonium and nitrate were determined colorimetrically. Oxidation of the substrate (ammonium chloride) was equivalent to the nitrification dynamics and was expressed as a percentage of the added nitrogen (3, 4, 45).

### Enumeration of culturable aerobic soil bacteria and soil fungi

Culturable heterotrophic soil bacteria and soil fungi were quantified by the plate-count method for viable cells. Appropriate dilutions of soil suspensions were spread on the surfaces of agar plates. The numbers of heterotrophic bacteria were determined on Standard I agar, supplemented with cycloheximide (400 μg mL^−1^) to exclude fungal growth. Soil fungi were enumerated on malt extract agar (5% [w/v] malt extract) supplemented with 0.02% (w/v) dl-lactic acid and chloramphenicol (100 μg mL^−1^) to exclude bacterial growth. Plates were incubated at 25°C for 48–72 h.

The microbial abundance of heterotrophic microorganisms was determined using culture-dependent methods. Since culturable cells may only represent ≤0.1–1% of the total microbial community in an environment (2, 41), culture-independent, molecular assays, such as profiling soil DNA, rRNA, or phospholipid fatty acids, are increasingly used (19, 33, 37, 43, 61). However, these methods were not well established at the beginning of the long-term monitoring in 1993 and were, thus, not included in this study. In order to allow for a comparison of the results obtained in 1993, 2000, and 2010, we consistently used the culture-dependent method as applied in 1993.

### Calculations and statistical treatment of data

Soil microbiological data are usually calculated on a dry mass basis. However, soil microbiological activity is highly dependent on the soil organic matter (SOM) content, which constitutes the basis of nutrition for heterotrophic soil microorganisms (45), while soil dry mass also contains material without relevance for microbial activity. Thus, the calculation of soil microbiological properties on a SOM basis is more appropriate for comparative studies of soils with highly differing SOM contents, as was the case in the present study. All measured parameters were calculated on a SOM basis.

Properties were determined for each soil sample (10 soil samples per site) in the spring and autumn of each sampling year, and the mean values of replicate determinations were used for statistical calculations (Statistica, version 8.0). Normal distributions were evaluated by the Kolmogorov-Smirnov-test. ANOVA was applied to determine whether the sampling year, sampling season, and/or the sampling site had a significant (*P*<0.05) effect on the soil microbiological parameters investigated. Kruskal-Wallis ANOVA and Mood’s median test were additionally applied for non-parametric data; however, these tests confirmed the results obtained by ANOVA. Multivariate statistics (principal component analysis) was applied to evaluate the relationship between the measured soil microbiological parameters and determining factors. Correlations between the determined properties in each site were tested by Spearman’s rank correlation coefficient since most of the data were non-parametric.

## Results and Discussion

### Climate conditions

The climate for the two studied sites is subalpine-continental with a solstitial pluvial curve (maximum values in summer and minimum values in winter). Site IT02 exhibited submediterranean influences with equinoctial pluvial regimes. This atmospheric condition, along with factors such as poor soil formation and low water retention capacity denoted occasional periods of summer aridity (1).

**Fig. 1:**
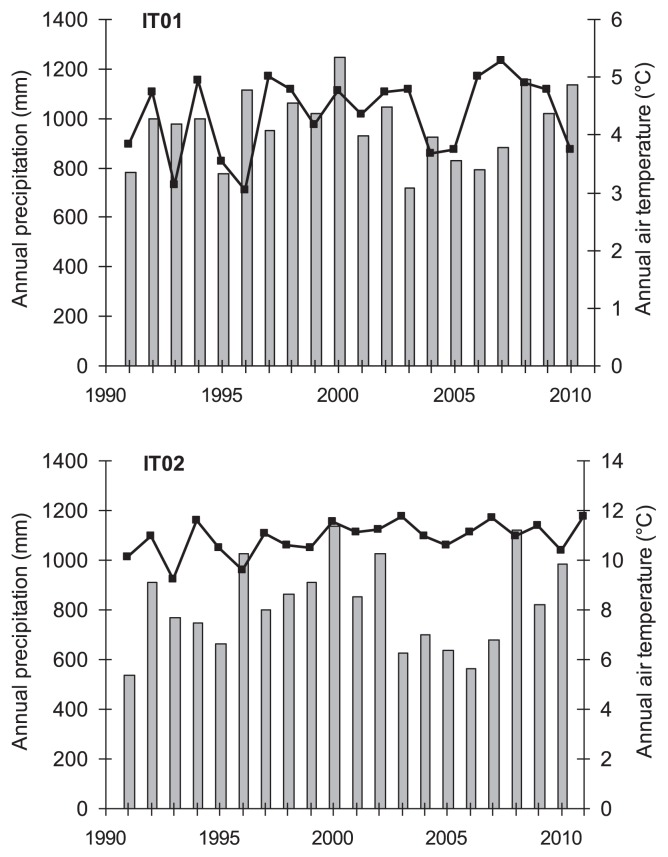
Climatograms showing mean annual air temperature (lines) and precipitation (columns) for the two study sites.

Mean annual air temperatures and precipitation at the two sites during the study period are shown in Fig. 1. Site IT01 was characterized by significantly (*P*<0.05) colder climatic conditions and larger temperature fluctuations than those at site IT02. Mean annual air temperatures were 4.3±0.7°C at site IT01 and 10.9±0.7°C at site IT02. Minimum and maximum air temperatures were 1.9±0.7°C and 7.1±0.9°C at site IT01, and were markedly higher at IT02 (5.8±0.5°C and 15.9±1.0°C). Site IT01 was also characterized by significantly higher annual precipitation (977±142 mm) than that at IT02 (830±174 mm).

Over the study period (1993–2010), an increase in the mean annual air temperature (+0.6°C and +0.8°C at IT01 and IT02, respectively) at both sites was calculated from trendlines. A 34-year-climatic series (1977–2011) at site IT02 even showed an increase in mean annual air temperatures over time from 10°C to 11.4°C (data not shown). An almost identical increase in temperature was reported at a site in northern Italy (6). An analysis of the climate regime in the period 2005–2009 over the Alpine area and northern Italy demonstrated the repeated occurrence of seasonal deviations (such as very cold winters, warm winters, rainy winters) over the last decade (6).

### Physical and chemical soil properties

Marked differences were observed in the nutrient contents of soil at the two studied sites. Soil from site IT01 contained significantly higher amounts of SOM (33%), C_org_ (19%), and nitrogen (0.8%) than that from site IT02 (12% SOM; 7% C_org_, 0.3% nitrogen); however, the C/N ratios were similar (Table 1). This is typical for subalpine forest soils, which contain high amounts of SOM in the A_h_ horizon due to slow litter degradation processes (especially at cold and humid sites). Soil from site IT01 was further characterized by its higher acidity (pH 3.3) than that from site IT02 (pH 4.1) (Table 1).

### Soil microbiological properties

#### Effect of the sampling year

The year of sampling had a significant effect on all investigated parameters, except nitrification (Tables 2 and 3). However, no consistent trend was observed among the investigated parameters. Phosphatase, sulfatase, respiration, SIR, and bacterial numbers were significantly higher in the second sampling period (2000) than in the first sampling period (1993), while the opposite was observed for dehydrogenase, xylanase, and protease as well as fungal numbers. Phosphatase, sulfatase and respiration were significantly lower in the third sampling period (2010) than in 2000, whereas no significant differences were observed in dehydrogenase, protease, and xylanase activities, SIR, and bacterial numbers between 2000 and 2010. These data can be explained by the climate conditions prevailing in each of the three sampling years: at both sites, air temperatures and precipitation were lower in 1993 than in 2000, while values recorded in 2010 were lower than those in 2000, but still higher than those in 1993 (Fig. 1). The constant decrease in fungal abundance over the study period could be attributed to a reduction in the C/N ratio because of accelerated litter degradation (due to increasing temperatures), which favored bacteria over fungi. C/N ratios decreased in spring from 24 (1993) to 22 (2000) and 21 (2010) at site IT01 and from 21 (1993) to 18 (2000) and 17 (2010) at site IT02. These decreases may have been due to a constant decrease in soil organic C with time, whereas N contents remained almost unchanged. Decreases in the relative abundance of fungi as a response to global warming have already been described (49, 58); however, these findings were obtained from experimental soil warming studies performed over short periods of up to 2 years, which makes it difficult to compare them with the results obtained in our study.

Overall, an increase in air temperatures of 0.6°C (IT01) and 0.8°C (IT02) over the study period of 17 years was noted at the two sites. A 35-year monitoring period (1977–2011) also revealed an increase in temperature of 1.4°C at site IT02. As already described above, the impact of increased temperatures on microbial communities and activities in forest soils has been evaluated in soil warming experiments conducted over a period of up to 2 years (15, 43, 49, 58); however, no studies have conducted experiments over a period of time that is similar to our long-term study. In addition, artificially warmed soils are characterized by a lower moisture content than that in controls without experimental warming, which is a serious drawback because soil moisture is the primary factor controlling decomposition rates in alpine forest soils (59).

#### Effect of seasonality

Season affected only a few of the measured microbiological parameters. Respiration and bacterial numbers were significantly higher in the spring than in the autumn, while the opposite was noted for the activities of xylanase and phosphatase. No significant differences were observed in any of the other parameters examined in this study between samples measured in the spring and autumn (Tables 2 and 3).

Significantly higher bacterial numbers in the spring than in the autumn, and the opposite trend for xylanase activity (higher activity in autumn than in spring), can be explained by the microorganisms involved in litter degradation. The highest amount of litter accumulates in autumn. This was clearly shown by comparing SOM contents in the spring and autumn: For example, in 2000, SOM contents at site IT01 were 36.5% and 80.3% in the spring and autumn, respectively; values recorded at site IT02 were 44.1% and 81.9%, respectively. Although litter contains components that are not easy to degrade, the degradation of these compounds by fungal cellulases and xylanases starts in autumn, and, consequently these activities are higher in the autumn than in the spring. After the snow melts in spring, easily degradable compounds are available and mainly converted by bacteria. Consequently, bacterial numbers are higher in the spring than in the autumn, which is consistent with the results of our study. Seasonal variations in microbial communities in forest soil were attributed to an altered resource supply, *i.e.*, winter communities had a higher capacity for the degradation of complex C substrates (plant cell walls), while summer communities were better adapted to utilize labile C sources (24). Seasonal variations in community structures and the function of alpine and subalpine soil microbial communities play a central role in biogeochemical cycling (16, 28). Forest soil microorganisms are characterized by high metabolic versatility and a capability to adapt to climatic changes associated with seasonality (8). At higher altitudes the decomposition of soil organic matter was shown to be more responsive to global warming because it is affected in a more sensitive (colder) temperature range than sites at lower altitudes (42). This has also been shown for proteolytic enzymes in forest soils (8).

### Comparison of sites

All measured parameters, except for nitrification and phosphatase activity, were significantly different between the two investigated sites; the subalpine site IT01 was generally characterized by significantly lower microbial activity and abundance than those observed at the submontane site IT02 (Tables 2 and 3).

This can be explained by the climatic conditions prevailing at the two sites. Site IT01, located at 1,737 m a.s.l., is exposed to significantly lower temperatures (lower by approximately 7°C on average over the study period), higher temperature fluctuations and higher precipitation (higher by 147 mm on average) than site IT02, which is located at 570 m a.s.l. High altitude ecosystems experience extreme temperature fluctuations with changing seasons and climates, higher precipitation, and a shorter vegetation period (25, 31). Due to these climatic conditions, microbial metabolism is slowed down and accumulating litter cannot be easily degraded, which results in the accumulation of raw humus.

### Effect of combinations of the sampling year, seasonality, and site

An evaluation of the effects of the combinations of the three independent factors (year, season, site) clearly indicated that a number of activities were influenced by the combination of sampling year and season (respiration, SIR, xylanase and phosphatase activities, and bacterial numbers) as well as by the combination of sampling year and site (SIR, dehydrogenase, xylanase, and protease activities, and fungal numbers). The combination of season and site only influenced bacterial numbers, while the combination of all three independent factors (year, season, and site) only had a significant effect on SIR (Table 3).

### Microbiological activities and the role of soil bacteria and soil fungi

**Fig. 2:**
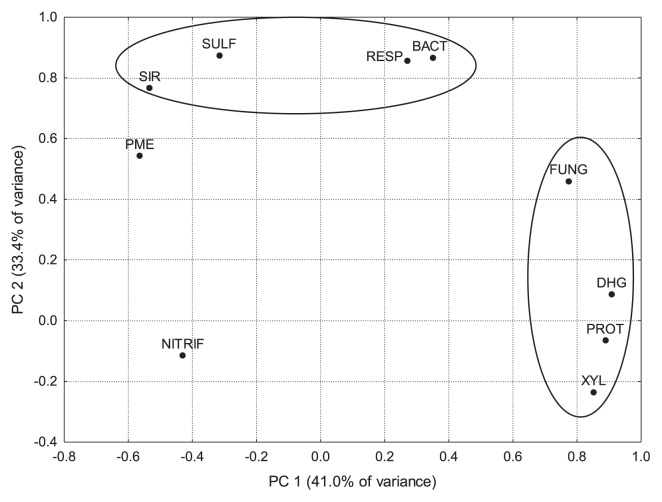
Loadings for the two first principal components (PC1 and PC2) of soil microbiological parameters determined in this study. Ellipses show parameters significantly (<0.7) contributing to PC1 or PC2. Abbreviations: RESP, respiration; SIR, substrate-induced respiration; DHG, dehydrogenase; XYL, xylanase; PROT, protease; PME, acidic phosphatase; SULF, sulfatase; NITRIF, nitrification; BACT, bacterial numbers; FUNG, fungal numbers.

Principal components (PC) were extracted for soil microbiological parameters measured over the whole study period at both sites in the spring and autumn. The first principal component (PC1) explained 41.0% of the variance in data while the second (PC2) accounted for 33.4%. Two clusters could be observed. PC1 was significantly (>0.7) determined by the activities of dehydrogenase, xylanase, and protease as well as fungal numbers (Fig. 2), and this could be explained by the important role of fungi in forest soils for primary litter degradation. Since litter mainly consists of xylanes and also contains a large amount of proteins, the activities of xylanases and proteases are required. In the course of fungal litter degradation, the C/N ratio is lowered, which in turn favors and allows for the activities of soil bacteria. PC2 was significantly determined by respiration, SIR, sulfatase activity, and bacterial numbers (Fig. 2). Bacteria in forest soils utilize the products released by fungal degradation and mineralize these products to CO_2_ and H_2_O, as demonstrated by the strong relationship between bacteria, respiration and SIR. Bacterial sulfatases degrade S-containing peptides and amino acids.

Nitrification and phosphatase activity did not contribute significantly to any of the clusters (to PC1 or PC2). Nitrification is mainly carried out by autotrophic bacteria (rarely and to a weak extent by heterotrophic bacteria) that were not detected by the culturable method for heterotrophic microorganisms used in the present study. Nitrification has mainly been attributed to acid-sensitive chemoautotrophic nitrifiers; however, this process has also been documented in acid forest soil (18, 29). Seasonal changes in ammonia-oxidizing microorganisms have also been observed in temperate forest soils (36).

**Fig. 3:**
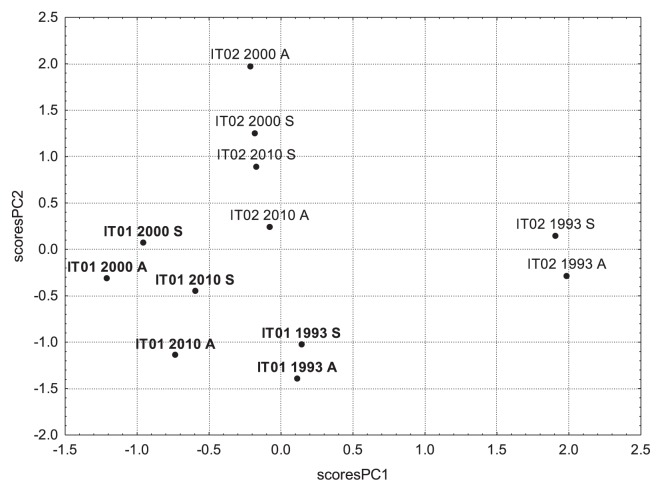
Score plot of PCA showing the separation of the two studied sites sampled in 1993, 2000, and 2010 in the spring (S) and in autumn (A).

When plotting the PC1 and PC2 scores for all soil samples analyzed, we obtained a clear separation between the two studied sites (Fig. 3). Within each site, PC2 factor scores were higher for values obtained in the spring than for those obtained in the autumn (except for site IT02 in the spring of 2000), which resembles the significantly higher microbial activities and bacterial abundance (significantly contributing to PC2) in the spring than in the autumn. Regarding the sampling years, factor scores were separated along PC1 and PC2. Variability over the study period was markedly higher in site IT02 than in site IT01. Especially, values obtained in 1993 were clearly separated from those obtained in 2000 and 2010, and this could be attributed to the higher temperature and precipitation recorded in 1993 than in 2000 at site IT02 (2.3°C, 372 mm) than at site IT01 (1.6°C, 266 mm).

Data obtained by PCA were confirmed by correlation analysis (Table 4) in which all data obtained at the two sites in the spring and autumn of 1993, 2000, and 2010 were included. The weak interaction of nitrification was confirmed by the absence of a correlation with most soil microbiological parameters. The positive correlation between xylanase and protease activities and fungal numbers, but the absence of a correlation between these activities and bacterial numbers confirmed the essential role of soil fungi with regards to these enzymes. On the other hand, sulfatase activity was positively correlated with bacterial numbers, but not with fungal numbers, which showed the dominating role of soil bacteria in soil metabolic processes such as the degradation of sulfur-containing compounds. The correlations observed between respiration, SIR, and dehydrogenase activity with bacterial as well as fungal numbers revealed that these microbiological parameters cannot be attributed exclusively to bacteria or fungi.

## Conclusions

At the two studied forest sites in the Italian Alps, increases in the mean annual air temperature of +0.6°C (IT01) and +0.8°C (IT02) over the monitoring period (1993–2010) were calculated from trendlines. A significant effect of the sampling year was detected on all microbiological properties studied (except nitrification), but with no consistent trend, whereas the effect of seasonality resulted in a significant response in a few parameters only. Soil microbial activities may be weakly correlated with climatic factors because they are typically estimated under optimized rather than *in situ* conditions (13). Enzyme activity measurements in soil are limited by the use of artificial reaction conditions and substrates and represent potential activities and may be insensitive to small temperature increases (21). However, they are a useful tool to compare the intensity, kind, and duration of specific effects (*e.g.*, environmental conditions) on the metabolic activity of soil (38, 39, 45, 57). In addition, the effects of climate change are likely to be driven by site-specific characteristics; therefore, it is important to consider the location-specific effects of climate change (11). The role of the microclimate in soil biological processes is often overlooked (47). The soil microclimate (temperature, moisture) is highly variable with time, which has been shown to cause temporal variations in microbial structures and activities (21, 23).

Biomonitoring sites also have the objective to quantify the variations between sites (26). We found significant differences in soil microbiological properties between the two investigated sites, *i.e.* microbial activity and abundance were lower in the subalpine site IT01 than in the submontane site IT02. These differences were mainly attributed to significant (altitude-dependent) differences in the climate conditions prevailing at these sites. However, other factors such as physical and chemical soil properties also play a role. Altitude, which strongly influences climate, was found to affect a number of soil biochemical properties (38). The relationship between altitude (increasing environmental harshness, *i.e.* lower annual temperature and soil nutrient contents) and a decrease in the microbial population size and diversity (28, 30, 32) as well as a decrease in a number of microbial activities (35, 44, 55) has been reported previously. However, other studies reported no consistent altitudinal trends regarding bacterial activity and community (14).

Therefore, further studies are warranted in order to obtain an insight into changing climate conditions on soil microorganisms. The combination of studies on soil microbial activity and diversity with special consideration of the soil microclimate should allow for a better understanding of the functional role of soil microorganisms in forest soils and will be the focus of future investigations.

## Figures and Tables

**Table 1:** Characteristics of the sites sampled in the late spring (May) and autumn (October) of 1993, 2000, and 2010.

Site	IT01 (Ritten)	IT02 (Montiggl)
Location	below Rittner Horn	above Small Lake Montiggl
Size	0.9 ha (confined)	0.9 ha (confined)
Altitude	1,737 m a.s.l.	570 m a.s.l.
Altitudinal vegetation belt	subalpine	submontane
Phytoclimate	*Piceetum*, subzone warm	*Castanetum*, subzone cold
Vegetation	Coniferous forests and shrubs	Mixed deciduous forests
	*Piceetum subalpinum*	*Quercetum pubescentis*
Bedrock	Silicate (porphyry)	Silicate (porphyry)
Soil type*	Haplic podsol	Dystric cambisol
Soil properties		
Sand (%; <2,000–63 μm)	48	36
Silt (%; <63–2 μm)	36	51
Clay (%; <2 μm)	16	13
CaCO_3_ (%)	<1%	<1%
pH (CaCl_2_)	3.3	4.1
SOM (%)	33	12
Corg (%)	19	7
C/N	24	21
N (mg 100 g^−1^)	826	327
P_2_O_5_ (mg 100 g^−1^)	4.1	1.6
K_2_O (mg 100 g^−1^)	21	11
Mg (mg 100 g^−1^)	16	14

* According to the soil classification of the Federal Republic of Germany (60)

**Table 2:** Mean values of the soil microbiological properties determined in this study. Different letters indicate significant (*P*<0.05) differences.

VARIATION		RESP	SIR	DHG	XYL	PROT	PME	SULF	NITRIF	BACT	FUNGI
Site	IT01	0.040 a	2157 a	77 a	3776 a	427 a	452 a	720 a	0.168 a	6.36 a	4.33 a
	IT02	0.057 b	3910 b	464 b	8238 b	817 b	391 a	1240 b	0.311 a	7.36 b	6.38 b
Year	1993	0.048 b	2047 a	548 b	521 b	130 b	99 a	352 a	0.033 a	6.68 a	6.23 a
	2000	0.059 c	3749 b	62 a	265 a	60 a	1097 c	1523 c	0.596 a	7.02 b	5.19 b
	2010	0.040 a	3305 b	202 a	130 a	75 a	167 b	1063 b	0.089 a	6.88 b	4.65 c
Season	Spring	0.052 b	2976 a	284 a	5065 a	1268 a	362 a	1094 a	0.111 a	6.96 b	5.33 a
	Autumn	0.046 a	3092 a	257 a	9950 b	776 a	481 b	865 a	0.368 a	6.77 a	5.38 a

Abbreviations: RESP, respiration (mg CO_2_ [g SOM * h]^−1^); SIR, substrate-induced respiration (mg CO_2_ [g SOM * h]^−1^); DHG, dehydrogenase (μg TPF [g SOM * 16 h]^−1^); XYL, xylanase (μg glucose [g SOM * 24 h]^−1^); PROT, protease (μg tyrosine [g SOM * 2 h]^−1^); PME, acidic phosphatase (μmol pNP [g SOM * h]^−1^); SULF, sulfatase (μmol pNP [g SOM * h]^−1^); NITRIF, nitrification (% N d^−1^); BACT, bacterial numbers (log cfu); FUNG, fungal numbers (log cfu).

**Table 3:** Effects of the study site, sampling season, and sampling year as well as their interactions on soil microbiological properties, as determined by ANOVA (Kruskal-Wallis ANOVA and Mood’s median test were additionally applied for non-parametric data and confirmed the results obtained by ANOVA).

Parameter	Effect of	Significant effect?		Result

		*P*-value	yes/no	
RESP	Year	**0.000**	yes	**2010 < 1993 < 2000**
	Season	**0.000**	yes	**Spring > Autumn**
	Site	**0.000**	yes	**IT01 < IT02**
	Site * year	0.504	no	
	Site * season	0.681	no	
	Year * season	**0.006**	yes	
	Site * year * season	0.148	no	
SIR	Year	**0.000**	yes	**1993 < 2000 = 2010**
	Season	0.588	no	Spring = Autumn
	Site	**0.000**	yes	**IT01 < IT02**
	Site * year	**0.019**	yes	
	Site * season	0.64	no	
	Year * season	**0.001**	yes	
	Site * year * season	**0.064**	yes	
DHG	Year	**0.000**	yes	**1993 > 2000 = 2010**
	Season	0.670	no	Spring = Autumn
	Site	**0.000**	yes	**IT01 < IT02**
	Site * year	**0.000**	yes	
	Site * season	0.761	no	
	Year * season	0.755	no	
	Site * year * season	0.224	no	
XYL	Year	**0.000**	yes	**1993 > 2000 = 2010**
	Season	**0.000**	yes	**Spring < Autumn**
	Site	**0.000**	yes	**IT01 < IT02**
	Site * year	**0.000**	yes	
	Site * season	0.256	no	
	Year * season	**0.000**	yes	
	Site * year * season	0.286	no	
PROT	Year	**0.000**	yes	**1993 > 2000 = 2010**
	Season	0.119	no	Spring = Autumn
	Site	**0.000**	yes	**IT01 < IT02**
	Site * year	**0.000**	yes	
	Site * season	0.303	no	
	Year * season	0.096	no	
	Site * year * season	0.356	no	
PME	Year	**0.000**	yes	**1993 < 2010 < 2000**
	Season	**0.000**	yes	**Spring < Autumn**
	Site	0.069	no	IT01 = IT02
	Site * year	0.159	no	
	Site * season	0.151	no	
	Year * season	.011	yes	
	Site * year * season	0.167	no	
SULF	Year	**0.000**	yes	**1993 < 2010 < 2000**
	Season	0.096	no	Spring = Autumn
	Site	**0.000**	yes	**IT01 < IT02**
	Site * year	0.135	no	
	Site * season	0.972	no	
	Year * season	0.135	no	
	Site * year * season	0.799	no	
NITRIF	Year	0.205	no	1993 = 2000 = 2010
	Season	0.365	no	Spring = Autumn
	Site	0.614	no	IT01 = IT02
	Site * year	0.761	no	
	Site * season	0.992	no	
	Year * season	0.210	no	
	Site * year * season	0.733	no	
BACTERIA	Year	**0.001**	yes	**1993 < 2000 = 2010**
	Season	**0.012**	yes	**Spring > Autumn**
	Site	**0.000**	yes	**IT01 < IT02**
	Site * year	0.245	no	
	Site * season	**0.040**	yes	
	Year * season	**0.006**	yes	
	Site * year * season	0.656	no	
FUNGI	Year	**0.000**	yes	**1993 > 2000 > 2010**
	Season	0.651	no	Spring = Autumn
	Site	**0.000**	yes	**IT01 < IT02**
	Site * year	**0.000**	yes	
	Site * season	0.055	no	
	Year * season	0.055	no	
	Site * year * season	0.082	no	

The significance level was *P*<0.05. Abbreviations: RESP, respiration; SIR, substrate-induced respiration; DHG, dehydrogenase; XYL, xylanase; PROT, protease; PME, acidic phosphatase; SULF, sulfatase; NITRIF, nitrification; BACTERIA, bacterial numbers; FUNGI, fungal numbers.

**Table 4:** Correlation matrix (Spearman rank order coefficients and significance level) between the soil microbiological parameters determined in this study

Parameter	RESP	SIR	DHG	XYL	PROT	NITRIF	PME	SULF	BACT
SIR	0.657 ***								
DHG	0.410 ***	0.415 ***							
XYL	0.295 **	−0.175 NS	0.279						
PROT	0.276 **	−0.052 NS	0.562 ***	0.754 ***					
NITRIF	0.034 NS	0.114 NS	0.009 NS	−0.186	−0.140 ***				
PME	0.259 **	0.504 ***	−0.250 **	−0.528 ***	−0.656 ***	0.052 NS			
SULF	0.451 ***	0.647 ***	0.292 **	−0.263 **	−0.094 NS	−0.042 NS	0.565 ***		
BACT	0.562 ***	0.651 ***	0.627 ***	0.061 NS	0.170 NS	−0.077 NS	0.214	0.523 ***	
FUNG	0.423 ***	0.249	0.633 ***	0.573 ***	0.538 ***	−0.177 NS	−0.263	−0.027 NS	0.614 ***

NS, not significant;^*^P<0.05;

** *P*<0.01;

*** *P*<0.001

Abbreviations: RESP, respiration; SIR, substrate-induced respiration; DHG, dehydrogenase; XYL, xylanase; PROT, protease; PME, acidic phosphatase; SULF, sulfatase; NITRIF, nitrification; BACT, bacterial numbers; FUNG, fungal numbers.
